# *In vivo* quantification of superficial cortical veins on susceptibility-weighted imaging with artificial intelligence image segmentation and the potential mechanism of human cognitive decline

**DOI:** 10.3389/fnagi.2025.1557397

**Published:** 2025-10-08

**Authors:** Qi Xie, Hai-Xia Xu, Ya-Jie Wang, Hui-Xian Chen, Xiao-Fang Tu, Peng-Peng Han, Jun Wu

**Affiliations:** ^1^Guangzhou First People’s Hospital, Guangzhou, China; ^2^Changsha Central Hospital, Changsha, Hunan, China; ^3^Guangzhou Institute of Software, Guangzhou, China

**Keywords:** magnetic resonance imaging, susceptibility weighted imaging, superficial cerebral veins, cognitive decline, image segmentation algorithm

## Abstract

**Objective:**

Changes in superficial cerebral veins (SCV) caused by different cognitive levels were observed using MR susceptibility-weighted imaging (MR-SWI) to explore the vascular mechanism underlying human brain aging and potential biomarkers of cognitive decline *in vivo*.

**Methods:**

Three hundred and sixty-four participants (184 males,180 females and aged 18–79 years) were included in this study. The quantitative features of SCVs in the cerebral hemispheres were collected via MR-SWI and were processed with an artificial intelligence (AI) image segmentation algorithm. The changes in the morphology and structure of the SCVs were analyzed with SPSS software.

**Results:**

The quantitative value of SCV were significantly greater in males than in females. In higher age groups, the total number of SCVs and the number of SCVs in the left and right cerebral hemispheres significantly decreased. The number of SCVs in hypertensive patients was significantly lower than that in non-hypertensive patients. Additionally, the diameter, curvature and length of SCVs in the right cerebral hemispheres were significantly lower in anemic patients than in non-anemic patients. The number and length of SCVs in the bilateral cerebral hemispheres were negatively correlated with the rate of cognitive abnormalities. Among tea drinkers in the youth group, the number of SCVs in both hemispheres were negatively correlated with total tau protein (T-tau), and the curvature of SCVs in the right hemisphere was negatively correlated with phospho-tau181(P-tau181) and T-tau concentrations in venous blood. There was a negative correlation between the T-tau concentration in venous blood and tea consumption. The curvature of SCVs in the right cerebral hemisphere had a significant impact on cognitive decline, with a strong positive correlation. The length of SCV in the right hemisphere of the brain had a significant negative correlation with cognitive decline, however, this correlation was relatively weak.

**Conclusion:**

The quantitative value of SCV was negatively correlated with cognitive decline. Daily tea consumption may have a positive impact on the quantitative features of SCVs in the young group. As SCVs are a component of the glymphatic system, their blood flow may affect the clearance of toxic proteins.

## Introduction

With the extension of the human lifespan, diseases related to cognitive decline, such as Alzheimer’s disease (AD), have become a serious public health problem (Soria Lopez et al., 2019; Villain and Michalon, 2024). Moreover, in the era of big data and informatization, many middle-aged and young people also experience early symptoms of cognitive decline (León Méndez et al., 2024). At present, the specific mechanism underlying cognitive decline is not fully understood, and the progress in its diagnosis and treatment of cognitive decline is slow. To date, there have been no significant breakthroughs in the clinical treatment of cognitive decline (Soria Lopez et al., 2019; Villain and Michalon, 2024).

Changes in cerebral hemodynamics play an important role in the pathological process of cognitive decline (Marley et al., 2021; Beura et al., 2024). Patients with cognitive decline often experience chronic cerebral ischemia or decreased cerebral blood flow, low perfusion leading to cerebral nutritional disorders, and long-term insufficiency of energy supply, all of which can have negative impacts on cognitive function (Beura et al., 2024; Ibrahim et al., 2021). Studies on cadaveric anatomy have shown that there are significantly fewer branches of superficial cerebral veins (SCVs) than of cerebral arteries, and one SCV needs to drain blood from 4 to 5 arteries (Hartmann et al., 2018; Ho et al., 2013). If an SCV is narrowed or occluded, it significantly increases the resistance of multiple upstream small arteries. Therefore, when pathological changes occur in an SCV, its compensatory ability to regulate local blood flow is much lower than that of arteries, which affects the blood flow perfusion function of upstream arteries in a coordinated manner. Additionally, brain health is closely related to the fluid flow dynamics that clear harmful waste, including flow around arteries, within the brain parenchyma, and around veins (Jiang-Xie et al., 2024). This system is regulated by vascular dynamics, maintenance of the surrounding vascular space, neural activity during sleep, and meningeal lymphatic drainage (Jiang-Xie et al., 2024). It can be inferred that in the neuroimaging exploration of the cerebrovascular mechanism of cognitive decline, in addition to observing the quantitative changes in cerebral blood perfusion from arteries *in vivo*, observing the morphology and structure of veins in vivo can be another entry point.

To date, some studies have shown that arterial spin labeling (ASL) perfusion of magnetic resonance imaging (MRI) for in vivo detection of quantitative changes in cerebral cortical arterial perfusion can provide information on arterial pathophysiology for early diagnosis of AD (Thropp et al., 2024). However, there have been few reports of similar research on the cerebral venous network.

Susceptibility-weighted imaging (SWI) by MR can effectively distinguish paramagnetic substances (deoxyhemoglobin) and is extremely sensitive for detecting venous blood. MR-SWI has become a clinically mature method for displaying human brain veins *in vivo* (Utrera Pérez et al., 2020). Our project team constructed an image segmentation algorithm model based on SWI for quantitative analysis of the cerebral venous network for the Han ethnic group, the main ethnic group in China in the preliminary research (Wang et al., 2023). This model can automatically identify and obtain the diameter, curvature, length, and number of SCVs in the bilaterial cerebral hemispheres. Our quantitative analysis of SCVs in three axial slices between the upper and lower edges of the lateral ventricle in the natural population revealed that males had better cognition than females did, and the number of SCVs per hemisphere was significantly greater in the male than in females. The number of SCVs was positively correlated with cognitive level (Wang et al., 2023).

On the basis of the above research (Wang et al., 2023), this study further quantitatively analyzed the SCV of the entire cerebral hemispheres of the participants, observing the morphological and structural changes in the SCV *in vivo* with different factors such as sex, age, cognitive status, chronic diseases, and diet, in order to explore the vascular mechanism of human brain aging and possible biomarkers of cognitive decline observed in vivo.

## Materials and methods

This study is a prospective observational analysis of community populations and was approved by the Ethics Committee of **** Hospital (No. K-2019-166-01). In accordance with the Declaration of Helsinki Declaration (revised in 2013), participants underwent medical examinations related to this study with their written informed consent.

### Participants

The subjects were recruited from the community and underwent the following process: basic information collection, objective neuropsychological scale assessment, cognitive assessment, noninvasive detection of limb arteriosclerosis, MR examination, and venous blood sampling and testing.

The basic information included the following: (1) genetic factors, such as age, sex, apolipoprotein E epsilon4(ApoE-ε4), and family history of AD; (2) external factors, such as years of education, occupation and diet (tobacco, alcohol, tea, fish, and coffee); (3) chronic diseases and accident, including history of surgical general anesthesia, history of brain trauma, history of poisoning, hypertension, diabetes, hyperlipidemia, arteriosclerosis, anemia, history of cerebrovascular accident, brain tumor, history of brain surgery, dementia, and chronic diseases that cause cognitive impairment.

The dietary habits of the participants were divided into the following categories: daily consumption, nondaily consumption, and nonconsumption for tobacco, alcohol, tea, and coffee. Fish consumption was divided into regular consumption (once a week or more), occasional consumption, and nonconsumption.

The objective neuropsychological scales included the Edinburgh Handedness Inventory (identifying right-handedness), the Hamilton Depression Scale (screening out depression), and the Hamilton Anxiety Scale (screening out anxiety disorders). The cognitive assessment scales used to evaluate participants’ memory, executive function, and language include the Auditory Verbal Learning Test- Hua Shan version (AVLT-H), the Shape Traits Test (STT), the Boston Naming Test (BNT), and the Animal Fluency Test (AFT).

The neuropsychological and cognitive scales for the older age individuals (≥ 60 years old) were as follows: (1) The Geriatric Depression Scale (GDS); (2) The Memory and Executive Screening (MES), which was used mild cognitive impairment (MCI); (3) The Functional Activities Questionnaire (FAQ), which was used to detect early or mild dementia patients.

The inclusion criteria for the participants were as follows: (1) Age ≥ 18 years of age and ≤ 79 years of age; male or female sex; (2) ≥ 7 years of education; (3) Han ethnicity; and (4) right-handedness. The exclusion criteria were as follows: (1) left-handedness or ambidexterity; (2) previous history of intracranial space occupying lesions, stroke, or cranial surgery; (3) There are central nervous system diseases that may cause cognitive impairment and related symptoms (such as Parkinson’s disease, depression, anxiety, or metabolic encephalopathy, etc.); (4) systemic diseases that may cause cognitive impairment, such as severe liver and kidney dysfunction, or thyroid dysfunction; (5) poor image quality on magnetic resonance imaging and failure to meet the requirements for data analysis; or (6) inability to cooperate in completing the relevant scale evaluation or withdrawal from the experimenter owing to unforeseen circumstances.

A total of 432 individuals entered the recruitment process. The following participants were excluded from the study: 38 individuals did not complete all the required scale assessments for the project, and their cognitive diagnosis could not be identified; 29 individuals had unsatisfactory MR image quality; and 1 individual had a clear history of cerebral infarction. Ultimately, 364 individuals were included, encompassing 184 males and 180 females aged 18–79 years (46.96 ± 13.74 years). A total of 226 participants underwent blood tests and had ApoE - *ε* 4 test results available, while 222 participants had quantitative results available for blood Amyloid-*β* 1–4 peptide (A β 1–42), total tau protein(T-tau), and phospho-tau181(P-tau181).

The age groups of the participants were divided into three subgroups according to the standards of the World Health Organization (WHO) (Ahmad et al., 2001): the young group (≤ 44 years old), the middle-aged group (45–59 years old), and the older age group (≥ 60 years old).

The recruited participants were divided into the following three categories according to the characteristics of their occupations (Human Resources Report, 2025): (1) physical professionals, whose work was dominated by physical activity and relied on muscle strength and physical exertion for the completion of tasks (e.g., laborers, farmers, and couriers); (2) mental professionals, whose work was centered around intellectual activities and involved solving problems through processes such as analysis, reasoning, and decision-making, emphasizing knowledge reserves and logical thinking abilities (e.g., programmers, non-physical education teachers, researchers, nonsurgical physicians, government department employees, and clerks); (León Méndez et al., 2024) mixed (mental plus physical) professionals, who work combined physical and mental characteristics, with practitioners possessing both physical dexterity and real-time judgment (e.g., surgeons, physical therapists, imaging technicians, nurses, maintenance engineers, physical education teachers, police officers, insurance salespeople, and domestic workers).

### Cognition data acquisition

Three professionally trained doctors (Xu H-X, Wang Y-J, Chen H-X) used the above scale to conduct a detailed evaluation and record of the participants in a quiet and comfortable environment. According to the cognitive diagnostic criteria for subjective cognitive decline (SCD), subjective cognitive decline plus (SCD-p) and MCI (Wang et al., 2020; Bondi et al., 2014; Han, 2018), the participants were divided into four groups including the normal cognition (NC) group, the SCD group, the SCD-p group and the MCI group.

The diagnostic criteria for NC are that the subject has no complaints or concerns about cognitive decline, and that there is no objective cognitive impairment.

The diagnosis of SCD was based on the standards proposed by Professor Han in the longitudinal study of subjective cognitive decline in multiple centers in China and the diagnosis and treatment strategies for subjective cognitive decline in the preclinical stage of AD in China (Wang et al., 2020; Han, 2018). In SCD, the subject has a chief complaint or subjective feeling of long-term or sustained cognitive decline, and the occurrence of this subjective feeling is not related to acute events. Moreover, for a subject to be classified as having SCD, the objective scale evaluation results of the participants must not meet the diagnostic criteria for MCI.

Among participants who met the diagnostic criteria for SCD, those who simultaneously meet one or more of the following conditions were included in the SCD-p group (Wang et al., 2020; Han, 2018): (1) the individual subjectively perceives a decrease in memory and does not have any other cognitive function decline; (2) the onset time is less than 5 years; (3) the age at onset is ≥ 60 years old; (4) the subject has concerns about cognitive decline; (5) the subject feels that his or her memory performance is worse than that of people of the same age; (6) cognitive decline is confirmed by informed individuals around the subject; (7) the genetic testing results show that the subject carries ApoE-*ε* 4; and (8) biochemical tests indicate the presence of AD biomarkers in the subject’s body. The fourth item is a necessary condition for diagnosing SCD -plus.

The diagnostic criteria adopted herein for MCI were the diagnostic criteria for early cognitive impairment proposed by Jak and Bondi (Bondi et al., 2014). If the participants have impaired scores on at least two indicators of the same cognitive domain, or have impaired scores on one test in each of the three cognitive domains, or score ≥ 9 on the FAQ, they are classified in the MCI group. The three cognitive domain assessments include memory (AVLT long delay and recognition), execution (STT-A and STT-B), and language function (AFT and BNT).

### Magnetic resonance data acquisition

#### MR examination

A Siemens Magnetom Skyra 3.0 T magnetic resonance scanner and a 32-channel head phased-array head coil were used. The subject underwent the following sequence of cranial examinations in the supine position, with the eyes closed and the head immobilized.

T1_mprage_3D_ sag_p2: Repetition time (TR) = 2,530 ms, echo time (TE) = 2.97 ms, inversion time (TI) = 1,100 ms, flipping angle (FA) = 7°, field of view (FOV) = 250 mm × 250 mm, matrix = 256 × 256, thickness = 1.0 mm, slice oversampling = 33.3%, orientation in the sagittal position, slices = 192, final voxel size = 1 × 1 × 1 mm^3^. Scan time = 4:30.

SWI_3D_tra_p2: TR = 28 ms, TE = 20 ms, thickness = 1 mm, slice oversampling = 11.1%, FA = 15°, axial orientation, FOV = 220 mm × 220 mm, matrix = 352 × 352, and final voxel size = 0.8 × 0.8 × 1 mm^3^. Scan time = 6:56.

#### SCV data acquisition from the cerebral hemispheres via SWI

The steps for the quantitative collection of cerebral hemisphere SCVs from SWI images were the same as those in previous studies conducted by our project team (Wang et al., 2023), and are briefly described below.

The raw data of the SWI Minimum Intensity Projection (MinIP) images are imported into the MicroDicom viewer.^1^ Axial images with a slice thickness of 20 mm, a gap of 1 mm, and a window width/window level of 40–65/30–45 were reconstructed to obtain clear display of the cerebral venous network. Continuous layers that can display bilateral cerebral hemisphere SCVs are selected and exported in JPG format. We randomly selected 20 participants’ MinIP images in JPG format and imported them into Labelme.^2^ After all visible SCV contours of the bilateral cerebral hemispheres were manually sketched one by one (Wang Y-J, Tu X-F), the image data were trained using the PSPNet image segmentation algorithm model based on SWI provided by the Software Institute of Guangzhou, Chinese Academy of Sciences (Han P–P, Tu X-F). The total number of training slices was 2000. Finally, a model with an average test set accuracy of 98.03% was obtained for use in this study (Tu X-F, Han P–P, Wu J).

**Figure 1:**
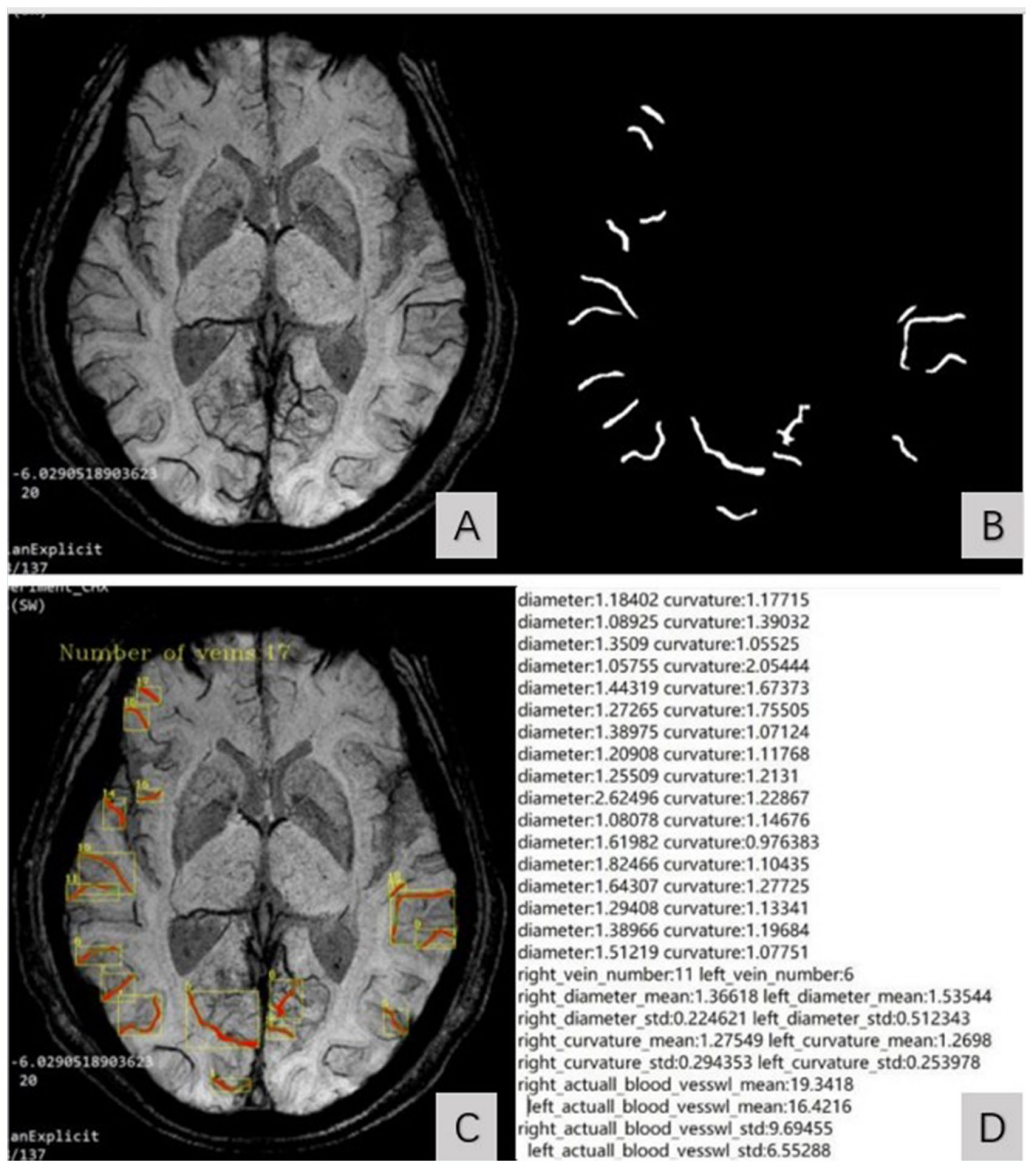
SCV image segmentation and recognition steps. **(A)** showed the original MinIP image. **(B)** showed the identified SCV of bilateral cerebral hemispheres. The identified SCVs were automatically superimposed on the original MinIP image **(C)** and generate the quantized value of the SCV at the detection slice **(D)**.

By inputting MinIP JPG images of all slices of the subject’s bilateral cerebral hemispheres into the model, the diameter, curvature, length, and number of SCVs in the bilateral cerebral hemisphere can be automatically identified and quantified. The output parameters include the diameter, curvature, length and number of SCVs displayed on the detection slice. The curvature of the SCV was defined as the ratio of the length of the venous vascular curve to the linear distance between two points on either end, indicating the tortuous degree of the SCV. The SCV quantification data of all slices in the bilateral cerebral hemispheres of each subject were automatically organized into an Excel table for statistical analysis. The process of the image segmentation algorithm model recognizing the SCV is shown in Figure 1.

### Noninvasive examination of limb arteriosclerosis

An Omron BP-203RPEIII from Japan was used to obtain data on the degree of arteriosclerosis in the limbs.

### Detection of ApoE-*ε*4 and toxic proteins related to cognitive decline in venous blood

After the participants completed the above neuropsychological and cognitive scale assessment, MRI examination and arteriosclerosis test, with their consent, a 6 mL of venous blood was collected from either side of the median cubital vein by a professional nurse within 24 h, and then placed into three vacuum blood collection vessels, respectively. After shaking, they were placed in a 0–4 °C portable refrigerator for refrigeration and sent for examination.

The detection of blood samples was completed by Kangshengda Medical Laboratory Co., Ltd., in Wuhan, Hubei Province. The method used was the same as that used in a previous study (Wang, 2022).

The ApoE-ε4 gene was detected by the second-generation gene sequencing technology. Combined with the nextseq550 gene sequencing platform of Illumina Company in the United States and its customized AD gene multiple PCR targeted capture panel, the target region of ApoE-ε 4 was captured at one time, and the constructed library was detected by the nextseq550 sequencer of the Illumina platform. An AngsDXTM custom AD panel for Illumina (Weizhi Gene Technology Co., Ltd., Hangzhou) was used as the kit.

The toxic proteins associated with AD were detected by enzyme-linked immunosorbent assay (ELISA). The ELISA kit was from Shanghai Boyan Biotechnology Co. Ltd., and the operation steps were performed in strict accordance with the instructions. Finally, the actual concentrations of Aβ 1–42, T-tau protein and P-tau181 were obtained.

### Statistical analysis

The collected data were input into the SPSS statistical software package (version 25.0; 174 IBM Corp., Armonk, NY, United States) for statistical analysis.

The counting data were analyzed using the chi square test. If the theoretical frequency in any grid is 1 ≤ t < 5, the chi square value needs to be continuously corrected. *p* < 0.05 was considered statistically significant.

The quantitative data are expressed as the mean ± standard error (M ± SE). First, if the sample size of the data was≥50, the Kolmogorov Smirnov (K-S) test was used to determine if the data followed a normal distribution; if the sample size was<50, the Shapiro Wilk test was used. For data that conformed to a normal distribution (*p* > 0.05), one-way analysis of variance (ANOVA) was used for comparisons of three or more groups, and Bonferroni (with equal variance) or Tamhane’s T2 (with uneven variance) tests were used for *post hoc* comparisons. Independent samples t-tests were used to compare normally distributed data between two groups. The nonparametric Kruskal–Wallis (K–W) test was used to compare nonnormally distributed data (*p* < 0.05 on the normality test) between three more groups, and Bonferroni correction (with equal variance) was used for post hoc comparisons. The Wilcoxon W test was used to compare nonnormally distributed data between two groups. *p* < 0.05 indicated a statistically significant difference.

Pearson’s correlation analysis (for normally distributed variables) and Spearman’s correlation analysis (for nonnormally distributed variables) were used to analyze the correlations between SCV values and various observation factors. *p* < 0.05 indicated a statistically significant difference.

A test of parallel lines was conducted to examine the cognitive status of 226 participants with venous blood ApoE-ε4 gene detection results. If *p* > 0.05, multinomial ordinal logistic regression was used to identify the impact of physiological factors that have a significant impact on SCV quantification values, ApoE-ε4 mutations, and SCV quantification values on cognition. *p* < 0.05 indicated a statistically significant difference.

## Results

### Baseline demographic characteristics and cognitive status distribution of the participants

After being grouped on the basis of genetic factors (age, sex, ApoE-ε4 and family history of AD), external factors (years of education, occupation, and diet including tobacco, wine, tea, fish, and coffee) and chronic diseases (history of general anesthesia, brain injury, poisoning, hypertension, diabetes, hyperlipidemia, arteriosclerosis and anemia), the distribution of demographic baseline data of 364 participants in different cognitive states is shown in Table 1. One subject was excluded due to uncertain occupation, 1 subject was excluded due to uncertain poisoning history, 2 participants were excluded due to uncertain family history, 3 participants were excluded due to uncertain diet, and 25 participants were excluded due to a lack of arteriosclerosis detection.

**Table 1:** Distribution of demographic baseline data (observation factors) of the participants in different cognitive states, *n* (%).

Observing Factors	Groups	Variables	Statistical value	NC	SCD	SCD-*p*	MCI	Sum
Genetic factors	Sex	Male	df = 3 *p* = 0.030	65 (35.3)	47 (25.5)	34 (18.5)	38 (20.7)	184
		Female		39 (21.7)	58 (32.2)	43 (23.9)	40 (22.2)	180
	Age	Youth,	df = 6 *p*<0.001	66 (40.2)	45 (27.4)	38 (23.2)	15 (9.1)	164
		Middle age		26 (21.8)	37 (31.1)	22 (18.5)	34 (28.6)	119
		Older age		12 (14.8)	21 (25.9)	19 (23.5)	29 (35.8)	81
	Family history of AD	Yes	df = 3 *p* = 0.175	12 (40.0)	9 (30.0)	7 (23.3)	2 (6.7)	30
		No		90 (27.1)	95 (28.6)	72 (21.7)	75 (22.6)	332
	AQE4 gene variation	Yes	df = 3 *p* = 0.338	12 (31.6)	14 (36.8)	8 (21.1)	4 (10.5)	38
		No		48 (25.5)	54 (28.7)	44 (23.4)	42 (22.3)	188
School education	Middle school	8-9 years	df = 9 *p*<0.001	9 (15.5)	15 (25.9)	10 (17.2)	24 (41.4)	58
	High school	10-12 years		20 (23.0)	23 (26.4)	17 (19.5)	27 (31.0)	87
	Undergraduate	14-16yeas		52 (31.1)	52 (31.1)	38 (22.8)	25 (15.0)	167
	Postgraduate	≥17yeas		23 (44.2)	14 (26.9)	14 (26.9)	1 (1.9)	52
Career	Occupation	Mental	df = 6 *p*<0.001	75 (31.8)	70 (29.7)	58 (24.6)	33 (14.0)	236
		Physical-Mental		16 (27.1)	19 (32.2)	6 (10.2)	18 (30.5)	59
		Physical		13 (19.1)	14 (20.6)	15 (22.1)	26 (38.2)	68
Eating habits	Smoking	Daily	df = 6 *p* = 0.893	17 (36.2)	10 (21.3)	10 (21.3)	10 (21.3)	47
		Nodaily		3 (27.3)	4 (36.4)	2 (18.2)	2 (18.2)	11
		Noconsumption		84 (27.7)	88 (29.0)	66 (21.8)	65 (21.5)	303
	Alcohol	Daily	df = 6 *p* = 0.956	6 (26.1)	7 (30.4)	4 (17.4)	6 (26.1)	23
		Nodaily		20 (27.4)	20 (27.4)	19 (26.0)	14 (19.2)	73
		Noconsumption		78 (29.4)	75 (28.3)	55 (20.8)	57 (21.5)	265
	Tea	Daily	df = 6 *p* = 0.865	41 (28.5)	46 (31.9)	29 (20.1)	28 (19.4)	144
		Nodaily		30 (30.3)	27 (27.3)	20 (20.2)	22 (22.2)	99
		Noconsumption		33 (28.0)	29 (24.6)	29 (24.6)	27 (22.9)	118
	Coffee	Daily	df = 6 *p* = 0.154	4 (22.2)	4 (22.2)	7 (38.9)	3 (16.7)	18
		Nodaily		15 (41.7)	9 (25.0)	9 (25.0)	3 (8.3)	36
		Noconsumption		85 (27.7)	89 (29.0)	62 (20.2)	71 (23.5)	307
	Fish consumption	Regular	df = 6 *p* = 0.091	31 (22.0)	39 (27.7)	40 (28.4)	31 (22.0)	141
		Occasional		16 (41.0)	12 (30.8)	5 (12.8)	6 (15.4)	39
		Noconsumption		57 (31.5)	51 (28.2)	33 (18.2)	40 (22.1)	181
chronic Diseases history	General anesthesia	Yes	df = 3 *p* = 0.52	22 (24.4)	36 (40.0)	20 (22.2)	12 (13.3)	90
		No		82 (29.9)	68 (24.8)	59 (21.5)	65 (23.7)	274
	TBI	Yes	df = 3 *p* = 0.646	3 (15.8)	6 (31.6)	5 (26.3)	5 (26.3)	19
		No		101 (29.3)	98 (28.4)	74 (21.4)	72 (20.9)	345
	Poisoning	Yes	df = 3 *p* = 0.535	2 (20.0)	2 (20.0)	4 (40.0)	2 (20.0)	10
		No		102 (28.9)	102 (28.9)	74 (21.0)	75 (21.2)	353
	Hypertension	Yes	df = 3 *p* = 0.470	13 (22.8)	21 (36.8)	12 (21.1)	11 (19.3)	57
		No		91 (29.6)	83 (27.0)	67 (21.8)	66 (21.5)	307
	Diabetes	Yes	df = 3 *p* = 0.856	6 (30.0)	7 (35.0)	3 (15.0)	4 (20.0)	20
		No		98 (28.5)	97 (28.2)	76 (22.1)	73 (21.2)	344
	Hyperlipidemia	Yes	df = 3 *p* = 0.162	6 (14.6)	16 (39.0)	9 (22)	10 (24.4)	41
		No		98 (30.3)	88 (27.2)	70 (21.7)	67 (20.7)	323
	Arteriosclerosis	Yes	df = 6 *p* = 0.196	20 (28.6)	17 (24.3)	11 (15.7)	22 (31.4)	70
		Mild		24 (23.8)	28 (27.7)	26 (25.7)	23 (22.8)	101
		No		52 (31.0)	49 (29.2)	39 (23.2)	28 (16.7)	168
	Anemia	Yes	df = 3 *p* = 0.869	6 (28.6)	7 (33.3)	5 (23.8)	3 (14.3)	21
		No		98 (28.6)	97 (28.3)	74 (21.6)	74 (21.6)	343

TBI, traumatic brain injury; AD, Alzheimer’s disease; NC, Normal cognition; SCD, subjective cognitive decline; SCD-p, SCD-plus; MCI, Mild cognitive impairment; df, degree of freedom. *χ*^2^ test.

There was no significant difference in the distribution of participants in different age groups between males and females [*χ*^2^ = 1.168, degrees of freedom(df) = 2, *p* = 0.558]. There was a significant difference in cognitive distribution between men and women (*χ*^2^ = 8.963, df = 3, *p* = 0.03; Table 1). The proportion of men with normal cognition (62.5%) was greater than that of women (37.5%) (*z* test, *p* < 0.05).

Cognition was lower at higher ages, and the difference was statistically significant (*χ*^2^ = 38.483, df = 6, *p* < 0.01; Table 1). The proportion of individuals with normal cognition in the young group (63.5%) was significantly greater than that in the middle-aged group (25.0%) and the older age group (11.5%) (*z* test, *p* < 0.05). The proportion of individuals with MCI in the middle-aged group (43.6%) and older age group (37.2%) was significantly greater than that in the young group (19.2%) (*z* test, *p* < 0.05).

The longer the length of schooling, the lower the incidence of cognitive decline (*χ*^2^ = 38.777, df = 9, *p* < 0.01; Table 1). The proportion of people with a bachelor degree or above with normal cognition (72.1%) was greater than that with high school education or below (27.9%) (*z*-test, *p* < 0.05). The proportion of individuals with MCI with a bachelor degree or above (33.7%) was lower than that with MCI with high school education or below (66.3%) (*z* test, *p* < 0.05).

There were significant differences in the cognitive assessments of participants from different professions (*χ*^2^ = 26.920, df = 6, *p* < 0.01; Table 1). The proportion of SCD-p in pure mental workers (73.4%) was greater than that in mental plus physical workers (7.6%) and pure physical workers (19.0%) (*z* test, *p* < 0.05). The proportion of MCI in pure mental workers (42.8%) was greater than that in mental plus physical workers (23.4%) and pure physical workers (33.8%) (*z* test, *p* < 0.05).

### Observation of the impact of demographic baseline indicators on cerebral hemisphere SCV

Statistical analysis revealed that age, sex, history of hypertension and anemia had statistically significant effects on SCV in the bilateral cerebral hemispheres (Table 2).

**Table 2:** The quantification value of detectable SCV in the cerebral hemispheres of different external factors ( $\bar{X}$ ±SE).

Group	Item	Number-total	Number-right	Number-left	Diameter-right	Diameter-left	Curvature-right	Curvature-left	Length-right	Length-left
Age (364)	Youth (164)	631.76 ± 15.44^**##^	323.12 ± 7.94^*##^	308.64 ± 7.84^*##^	1.4253 ± 0.0128^#^	1.3940 ± 0.0136	1.2078 ± 0.0106*^#^	1.1044 ± 0.0096^##^	16.439 ± 0.164*^##^	16.121 ± 0.176^##^
	Middle age (119)	571.92 ± 16.47^**^	293.79 ± 8.39^*^	278.13 ± 8.62^*^	1.4016 ± 0.0157	1.3428 ± 0.0149	1.2022 ± 0.0129^*^	1.0811 ± 0.0117	16.123 ± 0.199^*^	15.654 ± 0.204
	Older age (81)	530.25 ± 19.56^##^	273.49 ± 9.74^##^	256.75 ± 10.33^##^	1.3633 ± 0.0200^#^	1.3210 ± 0.0240	1.1486 ± 0.0173^#^	1.0830 ± 0.0174^##^	15.433 ± 0.262^##^	15.034 ± 0.292^##^
Sex (364)	Male (184)	642.94 ± 14.60^**^	327.01 ± 7.51^**^	315.93 ± 7.52^**^	1.4134 ± 0.0131	1.3849 ± 0.0130^**^	1.2043 ± 0.0111^*^	1.1063 ± 0.0098^**^	16.443 ± 0.166^**^	16.173 ± 0.174^**^
	Female (180)	535.09 ± 12.50^**^	277.42 ± 6.39^**^	257.67 ± 6.42^**^	1.3939 ± 0.0124	1.3366 ± 0.0139^**^	1.1811 ± 0.0102^*^	1.0593 ± 0.0099^**^	15.773 ± 0.159^**^	15.270 ± 0.171^**^
Career(363)	M (236)	590.56 ± 12.75	302.86 ± 6.53	287.70 ± 6.52	1.3984 ± 0.0.0115	1.3597 ± 0.0117	1.1836 ± 0.0098	1.0763 ± 0.0089	16.066 ± 0.151	15.691 ± 0.157
	M + *p* (59)	615.10 ± 25.64	312.76 ± 12.96	302.34 ± 13.30	1.4196 ± 0.0183	1.3722 ± 0.0190	1.2334 ± 0.0156	1.1162 ± 0.0118	16.439 ± 0.248	15.994 ± 0.210
	*p* (68)	566.13 ± 20.51	293.06 ± 10.24	273.07 ± 10.89	1.4089 ± 0.0221	1.3556 ± 0.0269	1.1913 ± 0.0168	1.0786 ± 0.0191	16.001 ± 0.255	15.623 ± 0.336
Hyper-tension(364)	No (307)	598.83 ± 10.85*	307.15 ± 5.57*	291.68 ± 5.57*	1.3995 ± 0.0095	1.3597 ± 0.0102	1.1946 ± 0.0081	1.0866 ± 0.0075	16.110 ± 0.123	15.777 ± 0.132
	Yes (57)	539.93 ± 25.32*	277.35 ± 12.29*	262.58 ± 13.51*	1.4266 ± 0.0259	1.3678 ± 0.0276	1.1830 ± 0.0212	1.0637 ± 0.0197	16.124 ± 0.339	15.456 ± 0.351
Anemia (364)	No (343)	592.11 ± 10.20	304.10 ± 5.22	288.01 ± 5.23	1.4116 ± 0.0091**	1.3665 ± 0.0098	1.1978 ± 0.0075*	1.0859 ± 0.0071	16.209 ± 0.115**	15.785 ± 0.125
	Yes (21)	548.71 ± 49.56	276.05 ± 22.89	272.67 ± 27.83	1.2753 ± 0.0401**	1.2710 ± 0.0433	1.1117 ± 0.0444*	1.0370 ± 0.0402	14.526 ± 0.628**	14.764 ± 0.634

SCV, superficial cerebral veins; M, mental professionals; P, physical professionals. *^OR#^*p* < 0.05; **^OR##^*p* < 0.01. Superscript * indicates the statistically significant difference between two adjacent groups. Superscript # indicates the statistically significant between the youth group and the older age.

**Figure 2:**
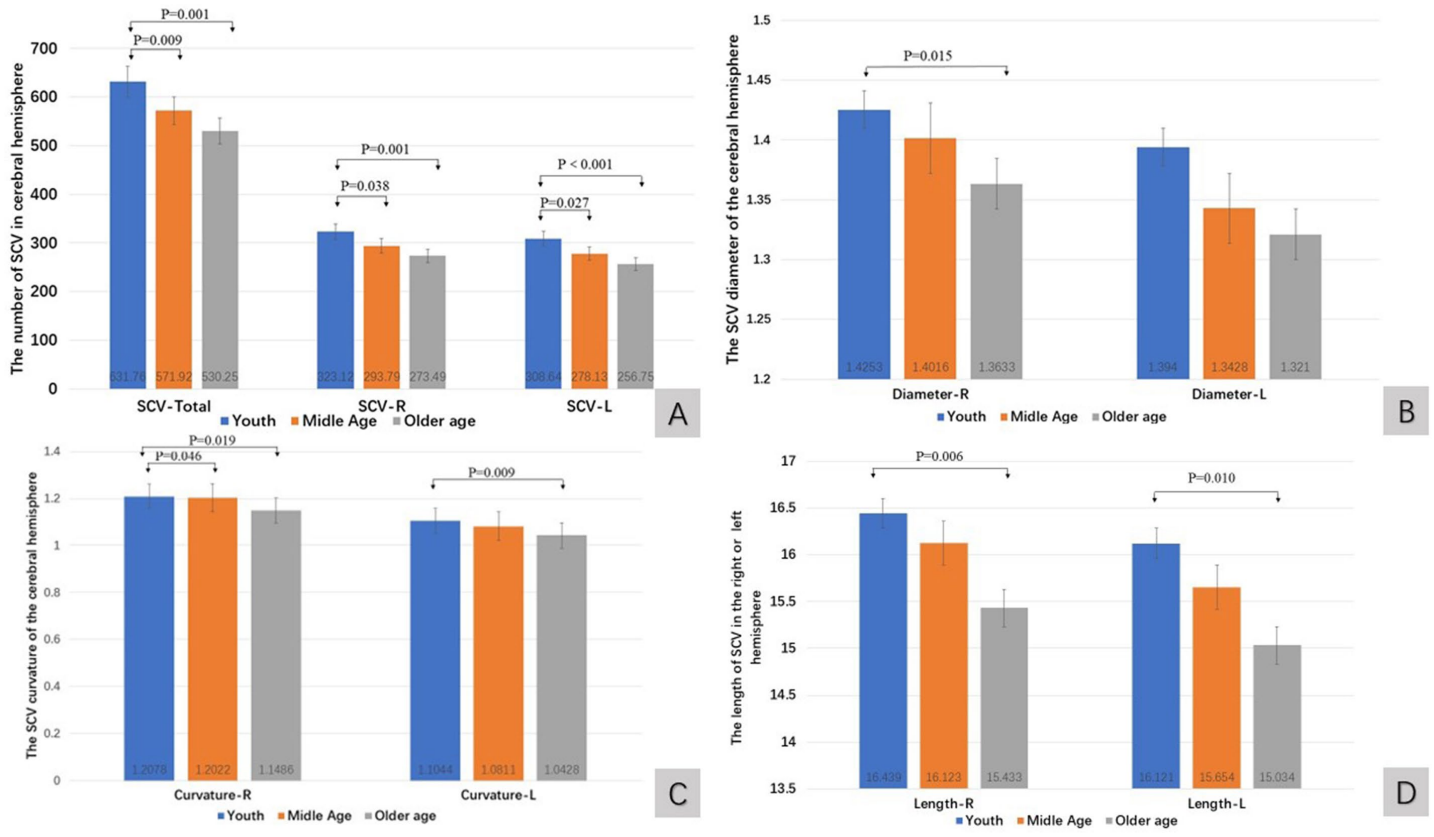
The quantitative value of SCV in cerebral hemisphere of age groups. Comparison of SCV number **(A)**, diameter **(B)**, curvature **(C)**, and length **(D)** of the cerebral hemispheres in different age groups. R: right cerebral hemisphere, L: left cerebral hemisphere.

In higher age groups, the quantitative value of the SCV in the human cerebral hemisphere tended to decrease (Figures 2A–D). The number of SCVs decreased significantly (K-W test, *p* < 0.01). The number of SCVs in both hemispheres, as well as in the left or right hemispheres, was significantly lower in the middle-aged and older age groups than in the young group (Bonferroni correction; Table 2; Figure 2A). The SCV diameter of the right hemisphere in the young group was greater than that in the older age group (Bonferroni, *p* = 0.015; Figure 2B), but there was no significant difference in the SCV diameter of the left hemisphere in pairwise comparisons of age groups (Bonferroni correction, *p* > 0.05; Table 2; Figure 2B). The SCV curvature of the right hemisphere in the older age group was smaller than that in the young group (Bonferroni correction, *p* = 0.019; Figure 2C) and the middle-aged group (Bonferroni correction, *p* = 0.046; Figure 2C). The SCV curvature of the left hemisphere in the older age group was smaller than that in the young group (Bonferroni correction, *p* = 0.009; Figure 2C). The length of the SCV in the right hemisphere in the older age group was shorter than that in the young group (Bonferroni correction, *p* = 0.006; Figure 2D). The length of the SCV in the left hemisphere in the older age group was shorter than that in the young group (Bonferroni, *p* = 0.010; Figure 2D).

**Figure 3:**
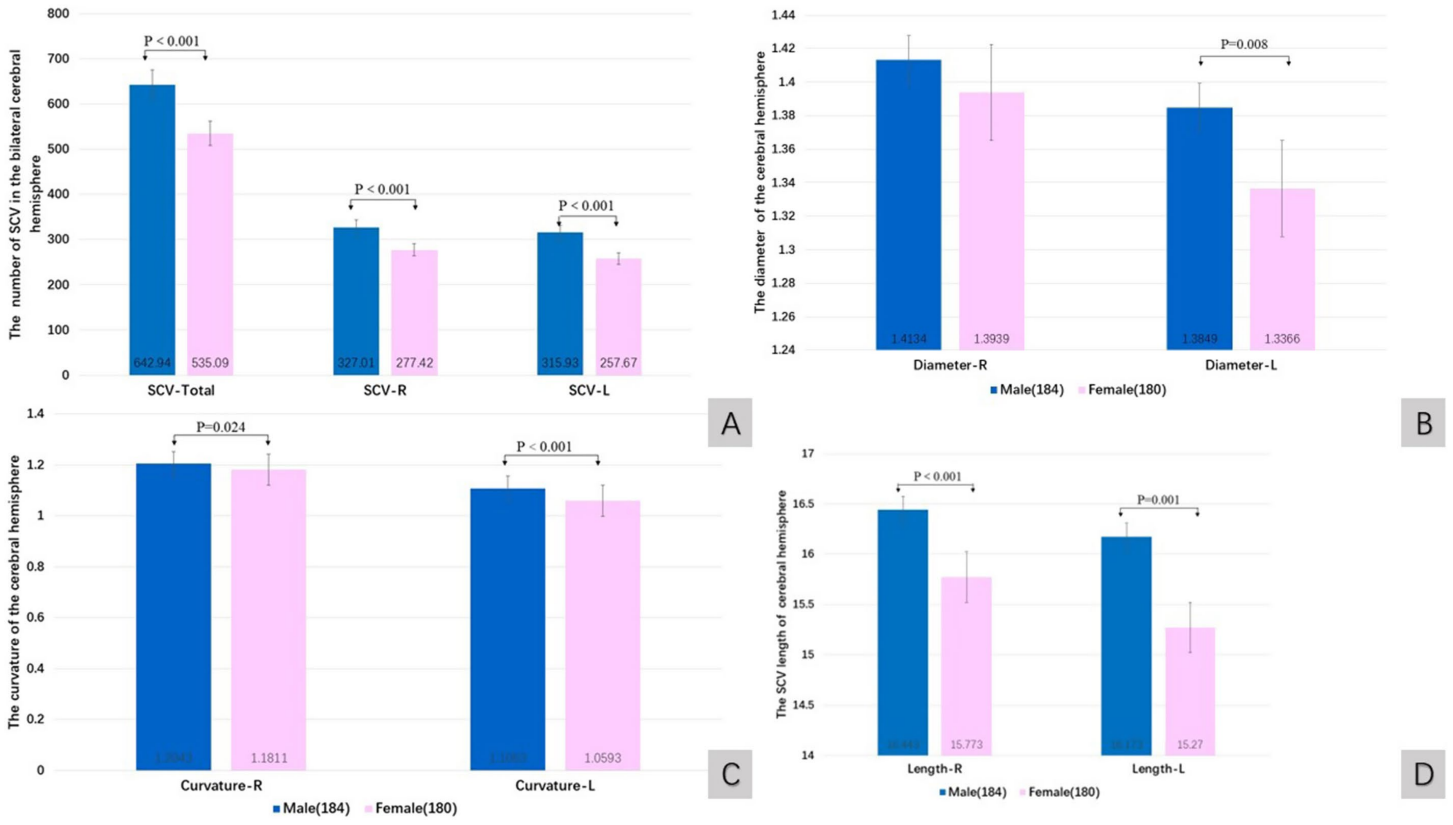
The quantitative value of SCV in cerebral hemisphere of sex groups. Comparison of SCV number **(A)**, diameter **(B)**, curvature **(C)**, and length **(D)** of the cerebral hemispheres in male and female groups. R: right cerebral hemisphere, L: left cerebral hemisphere.

The quantitative value of the SCV in males was greater than that in females. The total number of SCVs (Wilcoxon W, *p* < 0.001), the number of SCVs in the right cerebral hemisphere (Wilcoxon W, *p* < 0.001), the number of SCVs in the left cerebral hemisphere (Wilcoxon W, *p* < 0.001), the diameter of SCVs in the left cerebral hemisphere (Wilcoxon W, *p* = 0.008), the curvature of SCVs in the left cerebral hemisphere (Wilcoxon W, *p* < 0.001), the curvature of SCVs in the right cerebral hemisphere (Wilcoxon W, *p* = 0.024), the length of SCVs in the right cerebral hemisphere (Wilcoxon W, *p* < 0.001), and the length of SCVs in the left cerebral hemisphere (*t* test, *t* = 3.481, *p* = 0.001) were statistically significant differences (Figures 3A–D).

The quantitative value of SCV in mental plus physical professionals showed an increasing trend compared with those in pure mental or pure physical professionals (Table 2). However, there were no significant differences (K-W test, *p* > 0.05).

**Figure 4:**
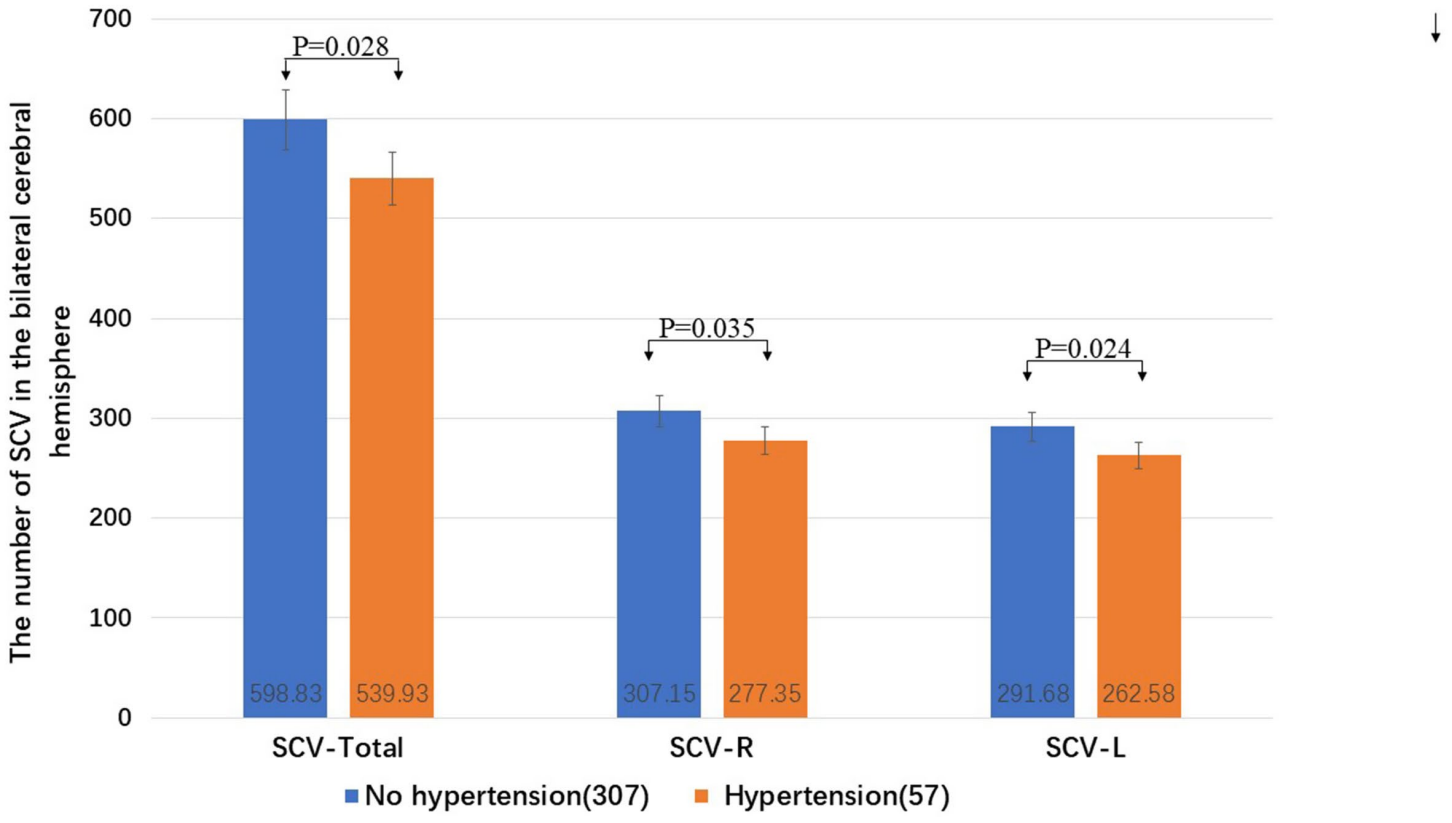
The influence of hypertension on the SCV number in the cerebral hemispheres. R: right cerebral hemisphere, L: left cerebral hemisphere.

The total number of SCVs in the bilateral cerebral hemisphere (Wilcoxon W, *p* = 0.028), the number of SCVs in the right cerebral hemisphere (Wilcoxon W, *p* = 0.035), and the number of SCVs in the left cerebral hemisphere (Wilcoxon W, *p* = 0.024) in patients with hypertension were significantly lower than those in patients without hypertension (Figure 4).

**Figure 5:**
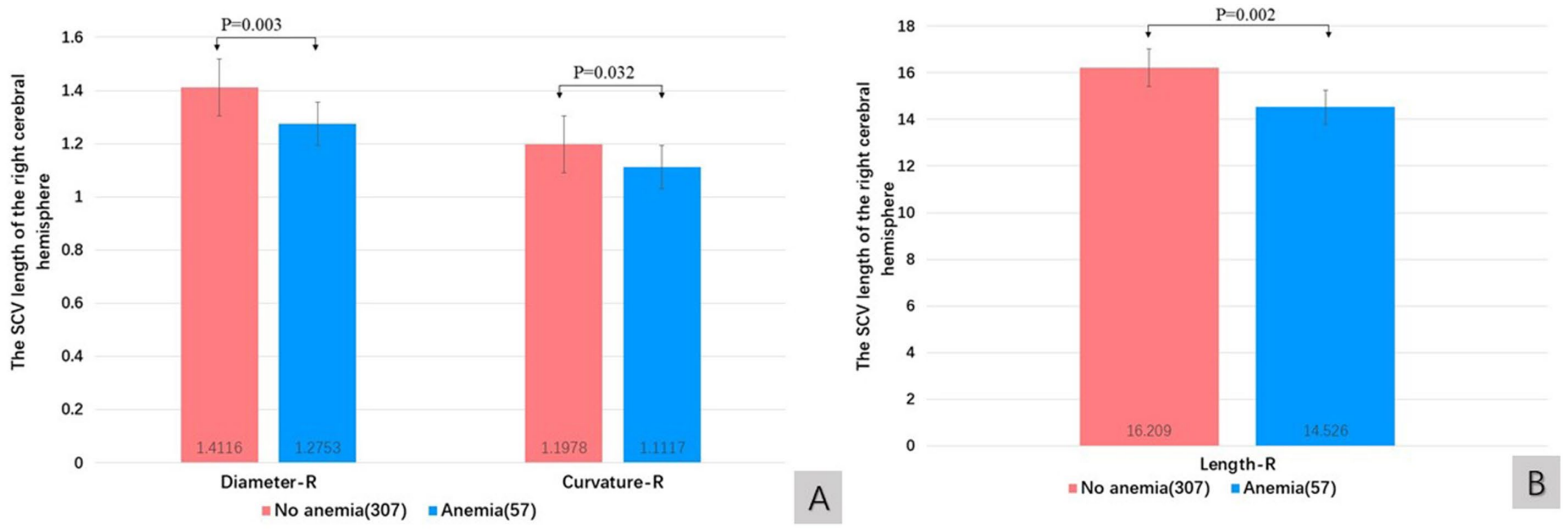
The influence of anemia on diameter and curvature **(A)**, and length **(B)** of SCV in cerebral hemispheres. R: right cerebral hemisphere, L: left cerebral hemisphere.

The quantitative value of the SCV tended to decrease in patients with anemia compared with those without anemia. There were significant differences in the SCV diameter of the right hemisphere (*t* test, *t* = 3.316, *p* = 0.003), the curvature of the right hemisphere (Wilcoxon W, *p* = 0.032) and the SCV length of the right hemisphere (Wilcoxon W, *p* = 0.002) between patients with anemia and those without anemia (Figures 5A,B).

### The impact of cerebral hemisphere SCV on cognitive status and its correlation in participants

The changes in the SCV in the cerebral hemisphere of participants in different cognitive states are shown in Table 3. The quantitative values of SCVs tended to decrease with cognitive decline. The length of the SCV in the left hemisphere decreased with cognitive decline (K-W test, *p* = 0.025). Compared with that in the normal cognitive group, the SCV length of the left cerebral hemisphere in the SCD group (Bonferroni correction, *p* = 0.025) was significantly shorter.

**Table 3:** SCV quantized values in cerebral hemisphere of participants with different cognition ( $\bar{X}$ ±SE).

Cognition	NC (104)	SCD (104)	SCD-*p* (79)	MCI (77)
Number-total	614.28 ± 16.54	580.59 ± 18.73	570.85 ± 21.10	587.71 ± 25.27
Number-right	314.52 ± 8.73	298.09 ± 9.40	293.09 ± 10.83	301.82 ± 12.57
Number-left	299.76 ± 8.34	282.50 ± 9.71	277.76 ± 10.86	285.90 ± 13.25
Diameter -right	1.4161 ± 0.0176	1.4062 ± 0.0148	1.3911 ± 0.0196	1.3970 ± 0.0212
Diameter -left	1.3820 ± 0.0183	1.3611 ± 0.0154	1.3397 ± 0.0215	1.3543 ± 0.0227
Curvature -right	1.2042 ± 0.0142	1.1975 ± 0.0127	1.1738 ± 0.0168	1.1906 ± 0.0176
Curvature -left	1.1019 ± 0.0132	1.0857 ± 0.0118	1.0633 ± 0.0147	1.0742 ± 0.0174
Length -right	16.424 ± 0.230	16.090 ± 0.181	15.905 ± 0.271	15.932 ± 0.259
Length -left	16.217 ± 0.245*	15.759 ± 0.218*	15.245 ± 0.246	15.514 ± 0.282

*p < 0.05. Superscript * indicates the statistically significant difference between NC group and SCD group.

Spearman’s correlation analysis revealed that cognitive abnormalities were negatively correlated with the total number of SCVs in the bilateral cerebral hemisphere (Spearman, *r* = −0.123, *p* = 0.019), the number of SCVs in the right cerebral hemisphere (Spearman, *r* = −0.112, *p* = 0.032) and left cerebral hemisphere (Spearman, *r* = −0.125, *p* = 0.017). Spearman’s correlation analysis also revealed that cognitive abnormalities were negatively correlated with the length of the SCV in the right hemisphere (Spearman, *r* = −0.124, *p* = 0.018) and the left hemisphere (Spearman, *r* = −0.129, *p* = 0.013).

### Regression model analysis of the impact of age, sex, ApoE *ε*4 variations, and SCV quantification values on cognition

The cognitive group of 226 participants whose ApoE - ε 4 testing results in vein blood were available passed test of parallel lines (*p* = 0.1625). The results of the multinomial ordinal logistic regression analysis are shown in Table 4. The Model Fitting Information indicated that the final model was significantly valid (*p* = 0.001). The model had a good fit (*p* = 0.984). The curvature of the right cerebral hemisphere SCV had a significant effect on cognitive decline (*p* = 0.002), indicating a strong positive correlation. The length of the SCV in the right hemisphere of the brain was significantly and negatively correlated cognitive decline (*p* = 0.038), and the influence was relatively weak. The age of the youth group has a significant negative correlation with cognitive decline (*p* < 0.001).

**Table 4:** Multinomial ordinal logistic regression of cognition level by independent variables.

Item			Estimate	Std. Error	Wald χ^2^	*p* value	95% Confidence Interval
Dependent Cognition threshold		Normal cognition	1.881	1.536	1.500	0.221	−1.129--4.892
		SCD	2.975	1.542	3.721	0.054	−0.048--5.998
		SCD-p	4.531	1.558	8.453	0.004	1.476--7.585
		MCI	(Reference group)				
Variables	Covariates SCV	Number-total	0.000	0.004	0.001	0.975	−0.007--0.007
		Number-right	−0.001	0.007	0.025	0.874	−0.014--0.012
		Number-left	(Reference group)				
		Diameter-right	−0.371	1.954	0.036	0.849	−4.201--3.459
		Diameter-left	2.963	2.044	2.102	0.147	−1.043--6.969
		Curvature-right	8.153	2.642	9.527	0.002	2.976--13.330
		Curvature-left	−3.946	3.091	1.630	0.202	−10.003--2.112
		Length-right	−0.336	0.162	4.295	0.038	−0.654-- -0.018
	Factors Genetic factor	Length-left	0.012	0.132	0.009	0.926	−0.247--0.271
		Male	0.030	0.263	0.013	0.911	−0.486--0.545
		Female	(Reference group)				
		Youth	−1.283	0.329	15.222	0.000	−1.928-- -0.639
		Middle age	−0.405	0.333	1.480	0.224	−1.058--0.248
		Older age	(Reference group)				
		No AQE4 variation	0.451	0.333	1.838	0.175	−0.201--1.103
		AQE4 variation	(Reference group)				

### Effect of daily tea consumption on SCV and toxic proteins in venous blood

The effects of tea consumption on the quantitative features of the SCV in the bilateral cerebral hemispheres and the levels of toxic proteins in venous blood are shown in Table 5.

**Table 5:** The effect of tea from different age groups on SCV and toxic proteins in the blood ( $\bar{X}$ ±SE).

Item (222)	Variables	Youth(99)			Middle age(72)			Older age(51)		
		No tea (36)	Occasional tea (33)	Daily tea (30)	No tea (17)	Occasional tea (14)	Daily tea (41)	No tea (20)	Occasional tea (14)	Daily tea (17)
The quanti-tative value of SCV in cerebral hemishe-res	Number-total	638.15 ± 26.59	584.63 ± 22.74	674.96 ± 29.44	545.58 ± 24.88	603.19 ± 29.04	574.29 ± 27.30	507.78 ± 28.42	530.47 ± 48.90	553.31 ± 31.91
	Number -right	326.45 ± 13.75	303.61 ± 11.52	340.48 ± 15.46	289.27 ± 12.97	302.00 ± 14.86	293.31 ± 13.92	261.56 ± 13.33	274.68 ± 25.82	284.62 ± 15.74
	Number -left	311.70 ± 13.38	281.02 ± 11.78^*^	334.48 ± 14.62^*^	256.30 ± 12.76	301.19 ± 15.83	280.98 ± 14.05	246.22 ± 15.56	255.79 ± 24.34	268.69 ± 17.23
	Diameter -right	1.4307 ± 0.0226	1.4240 ± 0.0232	1.4244 ± 0.0216	1.4008 ± 0.0264	1.3996 ± 0.0287	1.4010 ± 0.0254	1.3181 ± 0.0238	1.3933 ± 0.0461	1.3848 ± 0.0374
	Diameter -left	1.4152 ± 0.0251	1.3613 ± 0.0259	1.4069 ± 0.0195	1.3215 ± 0.0300	1.3575 ± 0.0278	1.3482 ± 0.0218	1.2577 ± 0.0316	1.3703 ± 0.0453	1.3555 ± 0.0474
	Curvature-right	1.1930 ± 0.0169	1.1922 ± 0.0175	1.2402 ± 0.0201	1.2357 ± 0.0168	1.2141 ± 0.0255	1.1787 ± 0.0213	1.1451 ± 0.0256	1.1299 ± 0.0381	1.1594 ± 0.0304
	Curvature -left	1.1008 ± 0.0166	1.0806 ± 0.0173	1.1320 ± 0.0158	1.0939 ± 0.0213	1.0917 ± 0.0198	1.0705 ± 0.0184	1.0233 ± 0.0258	1.0385 ± 0.0292	1.0668 ± 0.0346
	Length -right	16.378 ± 0.325	16.185 ± 0.250	16.806 ± 0.268	16.498 ± 0.266	15.982 ± 0.416	15.989 ± 0.324	15.179 ± 0.372	15.116 ± 0.552	15.835 ± 0.483
	Length -left	16.241 ± 0.327	15.630 ± 0.295	16.500 ± 0.292	15.775 ± 0.876	15.751 ± 0.396	15.531 ± 0.306	14.416 ± 0.385	15.338 ± 0.568	15.523 ± 0.578
Toxic proteins	Aβ1-42 ug/L	265.62 ± 39.45	237.90 ± 44.37	187.83 ± 38.76	175.36 ± 24.90	127.48 ± 18.37	163.58 ± 16.83	242.29 ± 47.24	122.60 ± 12.32	211.84 ± 52.75
	T-tau pg./ml	2307.04 ± 244.03	1828.53 ± 178.98	1731.85 ± 264.99	1483.88 ± 154.38	1500.07 ± 232.50	1697.26 ± 62.78	2204.28 ± 319.75	1390.32 ± 101.15	2114.06 ± 405.59
	P-tau181 ng/L	119.71 ± 21.21	83.23 ± 10.07	84.86 ± 20.15	130.13 ± 69.57	58.29 ± 10.01	72.81 ± 10.58	117.79 ± 25.94	64.08 ± 7.97	97.95 ± 30.29

SCV, superficial cerebral veins. ^*^p < 0.05.

In the young group, the number of SCVs in the left cerebral hemisphere (K-W test, *p* = 0.02), and the curvature of the SCV in the left cerebral hemisphere (K-W test, *p* = 0.037) significantly differed across the different tea drinking habits. However, only the number of SCVs in the left cerebral hemisphere in daily tea drinkers was significantly greater than that in no-daily tea drinkers (Bonferroni correction, *p* = 0.017).

Spearman’s correlation analysis revealed that the number of SCVs in the bilateral hemisphere was negatively correlated with the T-tau concentration in venous blood (Spearman, *r* = −0. 138, *p* = 0. 039), the curvature of the SCV in the right hemisphere was negatively correlated with P-tau181 (Spearman, *r* = −0. 156, *p* = 0. 020) and T-tau concentrations in venous blood (Spearman, *r* = −0.145, *p* = 0.029). Furthermore, the T-tau concentration was negatively correlated with tea consumption (Spearman, *r* = −0. 227, *p* = 0.024).

## Discussion

In this study, we strictly followed the diagnostic criteria proposed by Han and Jak/Bondi (Wang et al., 2020; Bondi et al., 2014; Han, 2018) to obtain clinical cognitive diagnoses of 364 participants, including NC, SCD, SCD-p, and MCI. Our observations revealed that the proportion of males with normal cognition was greater than that of females, indicating that males have better cognition than females do. Our research results also indicated that the proportion of cognitively normal individuals was significantly greater in the young group than thoes in the middle-aged and older age groups. The proportions of individuals with MCI in the middle-aged and older age groups were significantly greater than that in the young group. As the duration of education increased, the incidence of cognitive decline significantly decreased. The proportions of SCD-p and MCI in pure mental workers were greater than those in mixed mental and manual workers or pure manual workers.

The distribution of cognitive status in this group of people could suggest that age-related brain degeneration and sex are internal factors affecting cognitive decline. These findings are consistent with the results found by other researchers (Villain and Michalon, 2024; Au et al., 2017; Irvine et al., 2012). The external brain training generated by school education and vocational processes, combined with the sustained and regular physical exercise generated by physical labor, may help prevent cognitive decline in individuals.

In 2023, the Alzheimer’s Association (AA) proposed a definition of AD centered on Aβ, emphasizing the importance of pathological elevation of neurotoxic proteins such as Aβ and P-tau in the brain as specific neuropathological features of AD. On the basis of this definition of AD, preclinical AD accounts for approximately three times as many cases as symptomatic AD (Gustavsson et al., 2023). In recent years, research on the system that clears toxic substances from the brain has led to a new direction for exploring the mechanisms of cognitive decline (Nedergaard and Goldman, 2020; Iliff et al., 2012; Iliff et al., 2014; Xie et al., 2013; Rasmussen et al., 2018; Reeves et al., 2020; Eide and Ringstad, 2019; Taoka and Naganawa, 2021; Agarwal et al., 2019; Taoka and Naganawa, 2020; Naganawa and Taoka, 2022; Albayram et al., 2022; Patel et al., 2023).

On the basis of animal experimental research, Iliff et al. proposed the concept of the glymphatic system (GS) in the brain (Iliff et al., 2012). In this system, cerebrospinal fluid (CSF) enters the perivascular space (PVS) between the brain parenchyma and adjacent pia mater along the cerebral perforating arterioles, passes through the AQP4 channel on the endfeet of protrusions from astrocytes, and enters the brain interstitial fluid (ISF) in the brain parenchyma. After metabolites are exchanged, the fluid is drained to the meningeal lymphatic system through the paravenous PVS, thereby clearing waste (Iliff et al., 2012; Iliff et al., 2014).

Animal studies have shown that this system is more active during sleep, which suggests the importance and related mechanisms of increased GS activity during sleep in maintaining brain biological function (Xie et al., 2013). Clinical observations also revealed that functional impairment of the GS is related to the accumulation of abnormal proteins in neurodegenerative diseases such as AD and PD (Iliff et al., 2014; Rasmussen et al., 2018; Reeves et al., 2020; Eide and Ringstad, 2019).

In recent years, researchers have proposed a comprehensive concept of fluid dynamics in the central nervous system (CNS) (Jiang-Xie et al., 2024): the “nerve fluid” of the arterial, venous, CSF, and ISF systems. The brain parenchyma and four types of extracellular fluid chambers, namely arteries, veins, cerebrospinal fluid, and ISF, constitute the substance chambers that compete for space within the cranial cavity. Any structural and functional changes in these fluid chambers can alter brain fluid dynamics, potentially increasing intracranial pressure, affecting nerve cell respiration, and hindering the clearance of metabolic waste (Taoka and Naganawa, 2021; Agarwal et al., 2019; Taoka and Naganawa, 2020).

Therefore, the structure of the cerebral microcirculation is not limited to the capillaries at the arterial end and the venous network at the venous end, nor is it solely responsible for the exchange of oxygen and nutrients between arteries and veins. The cerebral microcirculation is also an important structure in the GS. The cerebral venous network acts as a bridge in the GS system. The MRI visualization of the structure and function of the cerebral GS is highly valuable for elucidating the pathogenesis of many neurodegenerative diseases, developing diagnostic criteria, and evaluating treatment outcomes. Researchers have conducted GS related MR visualization explorations of cerebral artery perfusion (Thropp et al., 2024), CSF (Naganawa and Taoka, 2022), and meningeal lymphatic drainage (Naganawa and Taoka, 2022; Albayram et al., 2022; Patel et al., 2023).

This study analyzed the SCV values of the entire cortex of the cerebral hemispheres on the basis of population baseline observation indicators, revealing that there were regular changes in the SCV values of both hemispheres. The number, curvature, and length of bilateral cerebral hemisphere SCVs, as well as the diameter of left cerebral hemisphere SCVs, were significantly greater in males than in females. In higher age groups, the quantitative values of SCVs in the human brain hemisphere showed a decreasing trend. The number, curvature, and length of the SCV in the bilateral cerebral hemispheres, as well as the SCV diameter in the right cerebral hemisphere, significantly decreased.

Owing to the lack of abundant energy storage, the brain is highly dependent on a continuous blood supply to maintain sufficient nutrient delivery and waste clearance (Santisteban et al., 2023). Venules have irregular lumens, with or without smooth muscle outside the endothelium, and their outer membranes are thin. The gap between endothelial cells in a postcapillary venule is relatively large, resulting in high permeability (Zedde and Pascarella, 2024). Therefore, we speculate that an increase in venous blood volume may lead to an increase in fluid flow into paravenous PVS and promote an increase in kinetic energy as the fluid flows into downstream meningeal lymphatic vessels.

The quantitative features of SCVs can reflect both upstream arterial blood perfusion and venous blood flow status (Pollock et al., 2008). On the basis of the above observations of cognition and SCV quantification values for different sexs and ages, we speculate that the decrease in SCV quantification values may reflect both a decrease in upstream arterial blood perfusion and a decrease in venous blood flow, leading to changes in fluid dynamics changes in the four extracellular fluid compartments of the brain parenchyma, which may cause a decrease in fluid flow and velocity in the paravenous PVS and a possible decrease in ability of the GS system to eliminate waste. The accumulation of toxic proteins such as Aβ and P-tau in the brain parenchyma may gradually increase, which may lead to adverse effects on the function of nerve cells and create a hidden dangers of cognitive decline. Therefore, the quantitative SCV features displayed by MR-SWI may reflect the waste clearance status of the GS and indirectly indicate abnormalities in the early stages of chronic brain injury with impaired cognitive function in humans.

This study also revealed that the quantitative values of cerebral SCV decreased with cognitive decline. Compared with that in the NC group, the length of SCVs in the left hemisphere in the SCD group was significantly reduced. The total number of SCVs in bilateral hemispheres, as well as in the right or left hemispheres, was negatively correlated with the occurrence of cognitive abnormalities. The SCV lengths in the right and left hemispheres were negatively correlated with the occurrence of cognitive abnormalities. The curvature of SCV in the right cerebral hemisphere was negatively correlated with the concentration of P-tau181 in venous blood. The above results further suggest that the quantitative features of SCVs in the bilateral hemispheres may reflect the pathophysiologic changes associated with cognitive decline earlier, making them possible biomarkers for predicting cognitive function earlier than clinical scales can do so.

In this group of cases, the total number of SCVs in the bilateral cerebral hemispheres was significantly reduced in hypertensive patients compared to non-hypertensive patients. This finding may suggest that chronic hypertension leads to a functional decrease in brain autoregulation to maintain constant cerebral CBF, resulting in decreased cerebral arterial perfusion (Santisteban et al., 2023). Anemia is also a risk factor for cognitive impairment (Tang and Sholzberg, 2024). This study revealed that the quantitative value of SCVs in anemic patients tended to decrease compared with those in nonanemic patients. Compared with those in patients without anemia, the diameter, curvature and length of SCVs in the right hemisphere of anemic patients were significantly reduced. The decrease in SCV volume caused by the above two chronic diseases may lead to a decrease in paravenous PVS fluid flow and velocity, which poses a hidden danger of toxic protein deposition in the brain and may be one of the pathological mechanisms leading to cognitive impairment.

Additionally, this study revealed that drinking tea had positive effects on young participants. The number of SCVs in the left hemisphere of daily tea drinkers was significantly greater than that in occasional tea drinkers. For daily tea drinkers, the number of SCVs in both cerebral hemispheres was negatively correlated with the T-tau concentration in venous blood, whereas the curvature of the right hemispheric SCVs was negatively correlated with the P-tau181 and T-tau concentrations in venous blood. The above results suggest that moderate daily tea consumption among young people may increase SCV blood flow, which may increase the clearance of toxic proteins.

The regression analysis in this study revealed that the curvature of the right cerebral hemisphere SCVs was strongly positively correlated cognitive decline, whereas the length of the right cerebral hemisphere SCVs was weakly negatively correlated cognitive decline. Age was significantly negatively correlated with cognitive decline in the young group.

Early cognitive decline is mainly characterized mainly by decreased memory, and the main executive center of human memory function is located in the right hemisphere of the brain(Tackett et al., 2014). We speculate that when memory decline occurs in the early stages of cognitive decline, the human brain may engage in self-rescue behavior to increase the blood supply to the right hemisphere of the brain, leading to an increase in local venous blood in the cortex related to memory (SCV curvature and length increase), while also improving toxin clearance. However, the microcirculation changes in the human brain are complex. An increase in SCV curvature in the right hemisphere may increase the clearance space and kinetic energy of the GS, and may also be indirect signs of increased local toxic proteins. The increase in SCV curvature in the right hemisphere may be a live monitoring sign of cerebral blood circulation changes during early cognitive decline. An increase in SCV length may reflect an increase in upstream arterial blood perfusion, which is beneficial for the functioning of the cerebral cortex and may be an indirect sign of cognitive recovery. As the young group ages, their experience increases, and their cognitive level significantly improves. The increase in SCV curvature in the right hemisphere of daily tea drinkers may also indicate the protective mechanism of tea on cerebral cortical blood circulation.

The brain SCV quantification model constructed using PSPNet in this study can efficiently obtain quantitative values such as the number, diameter, length, and curvature of SCV in bilateral cerebral hemispheres, with constant data, providing a reliable method for studying the cerebral venous network in the CNS. However, this study has the following limitations. First, the SCV quantification dataset in this study was derived from two-dimensional cross-sectional images, which cannot accurately capture the actual number of venous networks. Further model training is needed from a 3D dataset to obtain vein quantification that is closer to the actual situation. Second, the microcirculatory activity status of different functional zones in the brain varies, and the dynamic changes in the corresponding cerebral fluid pool need to be further evaluated according to brain functional zones. To evaluate the value of venous morphology, it is necessary to combine arterial perfusion, cortical functional status, and downstream meningeal lymphatic vessels in order to better illustrate the function of the cerebral venous network. Third, with respect to the effects of tea at different ages, the onset time of tea drinking, tea type, tea drinking habits, etc., in the middle-aged and older age groups were not accurately collected. It is necessary to conduct long-term follow-up on young participants with existing tea drinking habits and observe the relevant changes after they enter the middle-aged and older age group, in order to accurately evaluate the effects of tea on the SCVs in the brain and toxic substances in the blood of middle-aged and older age individuals. Fourth, with the exception of tea consumption, the sample distribution of other dietary habits was uneven. A larger sample size is needed for further observation and analysis.

The above limitations need to be further explored by us in collaboration with professionals from different disciplines, such as imaging technology, AI models, and their applications to the human brain.

In summary, cognitive abnormalities were negatively correlated with the quantitative value of SCVs in the bilateral cerebral hemisphere. The quantitative features of SCVs were higher in males than in females. In higher age groups, the quantitative features of SCVs decreases. The number of SCVs in the bilateral hemisphere was negatively correlated with the T-tau concentration in venous blood. The curvature of the SCV in the right hemisphere was negatively correlated with P-tau181 and T-tau concentrations in venous blood. The T-tau concentration in venous blood was negatively correlated with tea consumption. SCV may serve as a link in the GS system, and their blood flow may affect the clearance of toxic proteins. The quantitative evaluation of SCVs may be a potentially valuable direction for the early detection of neurodegenerative diseases and exploration of the mechanism of cognitive decline *in vivo*.

## Data Availability

Due to confidentiality reasons, the experimental data presented in this study cannot be shared publicly. Requests to access this data should be directed to the corresponding author where it can be provided after obtaining approval from the project research institution.

## Footnotes

^1^ https://www.microdicom.com/

^2^ https://github.com/wkentaro/labelme
