# Supplementary material for: In vivo quantification of superficial cortical veins on susceptibility-weighted imaging with artificial intelligence image segmentation and the potential mechanism of human cognitive decline
Source: Front Aging Neurosci. 2025 Oct 8;17:1557397. doi: 10.3389/fnagi.2025.1557397 (PMC12540377; doi:10.3389/fnagi.2025.1557397)
Supplement: Supplementary file 1 [file Data_Sheet_1.PDF]

Construction and application of PSPNet based semantic segmentation model for superficial cerebral veins

Part I. PSPNet image segmentation algorithm

1. SWI raw data processing of bilateral cerebral hemispheres

1.1 Selection of parameters for dataset reconstruction

The superficial cortical veins (SWI) are tortuous and have many branches, so this study used volumetric imaging to collect raw SWI data. When converting raw data into images required for model construction, comparative analysis of various reconstruction layer thicknesses through pre-experiments showed that a reconstruction layer thickness of 20mm and a window width/ window level of 40-65/30-45 had the best display effect on SCV. Therefore, this study used a layer thickness of 20mm and a layer spacing of 1mm as the reconstruction parameters for volumetric imaging data, and the reconstructed Minimum Intensity Projection (MinIP) was used as the image dataset for model construction.

1.2 Dataset Collection

Import the SWI raw volume data of the subjects into MicroDicom viewer （ https://www.microdicom.com/ ） to reconstruct a MinIP image with a layer thickness of 20mm, a layer spacing of 1mm, and a window width/position of 65-40/30-45 and obtain the best display of SCV. Select all layer images that can display SCV of cerebral hemispheres and export them in JPG format. Convert DICOM format images to JPG format, ensuring that the number of converted JPG images matches the number of layers selected for SWI scanning. Each image should fully display the image information of a slice and correspond to the corresponding scanning layer. By utilizing location information and scanning layers, specific layers and the

position of SCV can be quickly determined.

Randomly select 2000 SWI MinIP JPG format images of both cerebral hemispheres from 20 individuals as the dataset for model training.

2. Model construction

2.1 Data Preprocessing

Import the target level image into the open-source tool Labelme (URL: https://github.com/wkentaro/labelme） and manually depict all visible SCV contours. In the MinIP images reconstructed from SWI scanning, SCV displays the clearest linear black structures in the cortical regions that distinguish from the surrounding gray white brain parenchyma. The aim of this study is to identify SCVs and automatically measure their morphological parameters, so only the brain parenchymal regions need to be analyzed. The outer contours of the brain may interfere with the training of neural networks, so brain parenchymal extraction processing is required for the images.

2.2 Model Training

The experiment was conducted on the Ubuntu20.04 system with an Intel Inter Core CPU i7-12700K@3.0GHz , and RTX3090TI graphics card. Use the Python 2.0 framework and Python 3.10 version. In the experiment, the network structure of PSPNet is shown in the following figure (Figure 1), with ResNet101 as the backbone (some convolutional layers have been changed to dilated convolutions), and the most core part is pyramid pooling.

Pyramid pooling consists of four pooling layers of different scales, with kernel sizes of 1x1, 2x2, 3x3, and 6x6, respectively. After passing through four pooling layers of different scales, the input feature map is compressed by 1x1 convolution to 1/4 of the original channel number, and then restored to the original feature map size through bilinear interpolation. Finally, the original feature map is concatenated with pyramid

pooling features by channel to improve the accuracy of segmentation boundaries.

This study chose to iterate 1000 times and adopted a gradually decreasing method for learning rate to avoid falling into local optima, that is, adjusting the learning rate every 200 iterations. The input image size is fixed at 1024 × 1024, and the output data is the model classification prediction result. The main parameters for training deep learning models are as following. Model depth=101, Dropout ratio=0.5, batch size=128, whether to load pre trained model=true, number of training iterations (num_ epochs)=1000, learning rate=0.01.

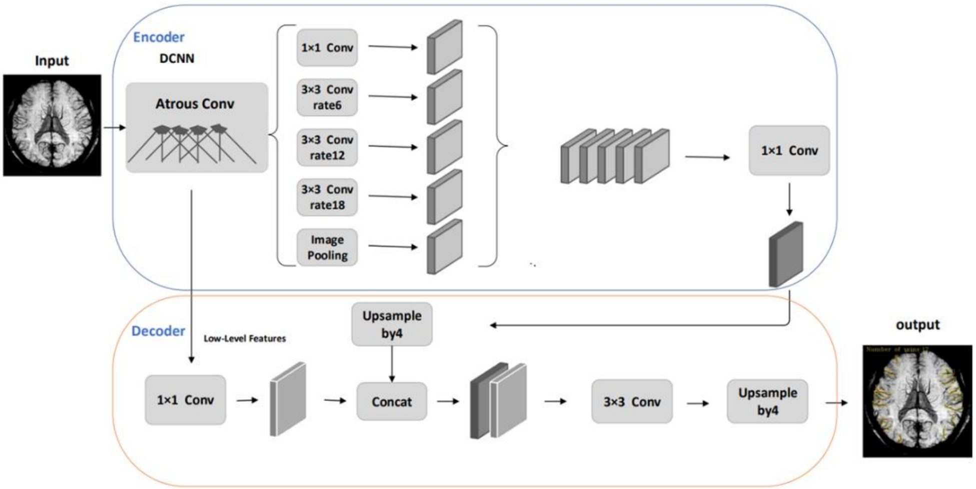

Fig. 1 Neural network model framework for automatic quantification of superficial cerebral veins.

2.3 Model Evaluation

Due to the limited amount of data, in order to improve the accuracy of the experiment, we adopted a four fold cross validation method to process 2000 sets of data. The data is randomly allocated to the training set, validation set, and test set in a ratio of 6:2:2. In the process of 4-fold cross validation, we used PSPNet network to train and validate on four different training and validation sets, and calculated the average accuracy of the four experiments. According to the results of four fold cross validation, the average training set accuracy of the four experiments was 98.19%, the average Verification set accuracy was 98.02%, and the average test set accuracy reached 98.03% (see Table 1)

Tab.1 The optimal model in each group of experimental indicators（%）

Cross experiment number	First group	Second group	Third group	Fourth group	Average values
Training set accuracy	98.11	98.01	98.21	98.40	98.19
Verification set accuracy	97.74	98.10	98.01	98.22	98.02
Test set accuracy	98.00%	97.90%	98.17%	98.07%	98.03%

3. Model application

By selecting MinIP JPG images of all slices of the subject's cerebral hemisphere and placing them in the model, the diameter, vascular curvature, length, and number of SCV in cerebral hemisphere can be automatically recognized and quantified. The output parameters include the diameter, curvature, length, and number of SCV at the detection slice. Automatically generate an EXCEL table of SCV quantification values for all detection slices and the sum of each detection value. Organize the quantitative data of SCVs in bilateral cerebral hemispheres of each subject separately for statistical analysis. The process and data presentation results of the image segmentation algorithm model for identifying SWI superficial cerebral veins are shown in Figure 2.

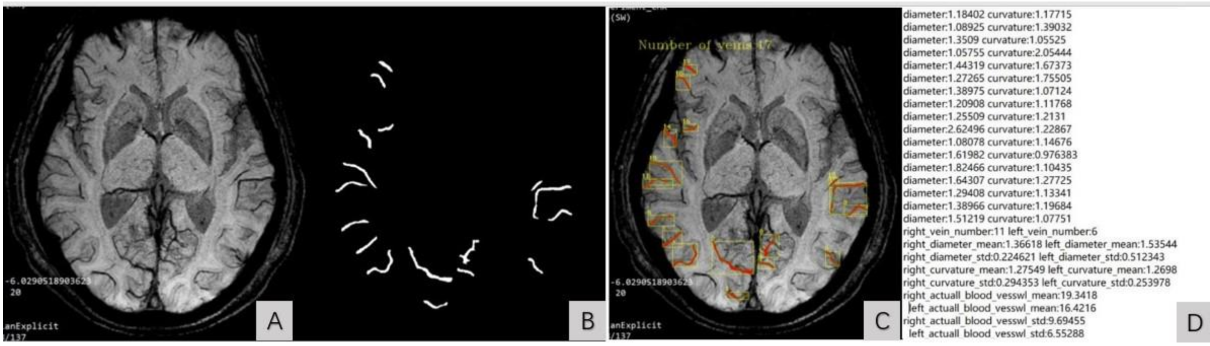

Figure2. SCV image segmentation and recognition steps. Figure A showed the original MinIP image. Figure B showed the identified SCV of bilateral cerebral hemispheres. The identified SCVs were automatically superimposed on the original

MinIP image (C) and generate the quantized value of the SCV at the detection slice(D).

Part II. Definition and calculation formula of detection indicators

The length of a SCV is the curve distance between its two ends.

Math.sqrt((x2 - x1)**2 + (y2 - y1)**2).

The curvature of SCV was defined as the curve length at both ends of the SCV divided by the straight length.

The mathematical formulas involved： k=|y^" |/ 〖(1+y^'2)〗 ^(3/2)

The diameter was defined as the ratio of the area of the superficial cerebral veins to the length of the tortuous length.

The mathematical formula involved: For any two points (x_i, y_i) and (x_j, y_j) in a point set, the Euclidean distance between them can be expressed as:

$$d = \sqrt{(x_j - x_i)^2 + (y_j - y_i)^2}$$
